# The simultaneous assessment of glycosylated hemoglobin, fasting plasma glucose and oral glucose tolerance test does not improve the detection of type 2 diabetes mellitus in Colombian adults

**DOI:** 10.1371/journal.pone.0194446

**Published:** 2018-04-13

**Authors:** Jose Lopez-Lopez, Jennifer Garay, Edwin Wandurraga, Paul A. Camacho, Fernando Higuera-Escalante, Daniel Cohen, Patricio Lopez-Jaramillo

**Affiliations:** 1 Internal Medicine Residence Program, Universidad Autonoma de Bucaramanga (UNAB), Bucaramanga, Colombia; 2 Endocrinology Service, Fundación Oftalmológica de Santander—Clínica Carlos Ardila Lulle (FOSCAL), Floridablanca, Colombia; 3 Research Department, Fundacion Oftalmologica de Santander (FOSCAL), Floridablanca, Colombia; 4 Facultad de ciencias de la Salud, Universidad Autonoma de Bucaramanga (UNAB), Bucaramanga, Colombia; 5 Higuera Escalante Laboratory, Floridablanca, Colombia; 6 MASIRA Institute, Facultad de Salud, Universidad de Santander (UDES), Bucaramanga, Colombia; 7 Facultad de ciencias de la Salud, Universidad Autonoma de Bucaramanga (UNAB), Bucaramanga, Colombia; 8 Facultad de Ciencias de la Salud Eugenio Espejo, Universidad Tecnológica Equinoccial, Quito, Ecuador; University of Catanzaro, ITALY

## Abstract

**Introduction:**

Guidelines recommend early detection of type 2 diabetes mellitus (DM2). The objective of the present study was to evaluate the capacity to identify DM2 in subjects that were screened for DM2 simultaneously with all three of the tests recommended—fasting plasma glucose (FPG), glycosylated hemoglobin (HbA1c) and a 2-hour post 75-g oral glucose tolerance test (OGTT).

**Methods and results:**

The present analysis of an anonymous database of 1113 adults from a reference clinical laboratory in Bucaramanga, Colombia, was an observational, descriptive, cross-sectional secondary source study. 259 individuals met at least one of the criteria for DM2: FPG ≥ 126mg/dL (7.0mmol/L), HbA1c ≥ 6.5% (48mmol/mol) and OGTT ≥ 200mg/dL (11.1mmol/L). 30 subjects (2.7%) were diabetic according to FPG, 56 subjects (5.0%) by HbA1c and 250 subjects (22.5%) by OGTT. In total 259 subjects (23.3% [IC 95%: 20.7%– 25.8%] were diabetic either by FPG, OGTT or HbA1c.

**Discussion:**

The largest number of patients were identified as diabetic with the OGTT. The combination of two or three tests did not increase the detection of new cases of DM2. Our findings suggest that routinely requesting FPG, OGTT and HbA1c at the same time may be inappropriate in at risk individuals, as this approach does not significantly improve the diagnostic capacity compared to using FPG+OGTT and substantially increases the financial burden on the health system, insurers or individual.

## Background

The prevalence of type 2 diabetes mellitus (DM2) continues to increase globally, with approximately 415 million people currently estimated to be suffering this disease [1]. DM2 is among the leading causes of morbidity and mortality due to its association with mortality from cardiovascular diseases [1, 2]. In addition, DM2 produces a high degree of disability and is considered a high cost disease [3, 4], placing a significant burden on health care systems.

Guidelines recommend the early detection of DM2 in order to implement an adequate control and delay the onset of complications [5, 6]. According to the American Diabetes Association (ADA) guidelines, an individual is diagnosed as DM2 if they meet any one of the following criteria: fasting plasma glucose (FPG) ≥ 126 mg/dl or 2-hour 75-g oral glucose tolerance test (OGTT) ≥ 200 mg/dl or hemoglobin HbA1c (HbA1c) ≥ 6.5%.

As all of these tests meet the criteria to be defined as gold standard, but the results of a single test could be misleading due to a number of factors such as reproducibility of the test, ethnicity, hemoglobinopathies, red blood cell turnover, physicians often elect send patients for all three tests simultaneously. The ADA guideline has taken this issue in to account and recommends that when two tests are performed and just one is above the cut-off point, the same test should be repeated to confirm the diagnoses, rather than performing an additional screening test [6]. Although the risk factors [7] and the criteria for the diagnosis of DM2 are well defined [5,6], there is ongoing debate as to whether these criteria are universally associated with the future risk of vascular complications [8]. Furthermore, a recent study found that based on the current cut-points, HbA1c, had a poor capacity to detect DM2 in US adults relative to OGTT and FPG [9]. Given the expense of conducting all three tests and the fact that in Colombia HbA1c is the most expensive of these, we aimed to determine the capacity of FPG, OGTT and HbA1c to detect DM2 in Colombian adults, and if their simultaneous assessment increases the diagnosis of DM2 according to the criteria of the Colombian guidelines for diabetes and the American Diabetes Association (ADA) [5, 6].

## Methods

The present analysis is an observational, descriptive, cross-sectional. The information was collected from the anonymous database of the *Laboratorio Higuera Escalante* (Floridablanca, Santander, COL), associated with the *Fundación Oftalmologica de Santander* (Floridablanca, Santander, COL). The clinical laboratory maintained the confidentiality of the subjects. The institutional review board of the *Fundación Oftalmologica de Santander* (FOSCAL) approved the study. The data obtained were the results of the examination of a population of aged over 18 years, who were screened for FPG, OGTT and HbA1c, in a single visit to the laboratory between January 2015 and February 2017.

A FPG was taken in the morning following an 8 hour overnight fast. An OGTT was taken two hours after the administration of 75 grams of glucose diluted in 300 ml of water, using the venipuncture technique with a safety system. Blood glucose was determined with the reference enzyme method using hexokinase. HbA1c was determined using the turbidimetric inhibition immunoassay (TINA) technique for hemolyzed whole blood using HbA1c3 (Tina-quant Hemoglobin HbA1c) and HbA1cD2 (Hemolyzing Reagent).

A descriptive analysis was carried out to estimate central tendency and dispersion according to the frequency distribution of the data. Categorical variables were summarized with absolute values and proportions, and their corresponding 95% confidence interval. We evaluated age related trends in FPG, HbA1c and OGTT using the Wilcoxon rank-sum test. Weighted Cohen's Kappa Coefficient was calculated to analyze the agreement between FPG and HbA1c with OGTT. Data was analyzed using STATA VE 12.0 software (StataCorp. Stata Statistical Software: Release 12. College Station, TX: StataCorp LP) with a significance level of 5%.

## Results

Between January 2015 and February 2017, the *Laboratorio Higuera Escalante* performed 214,406 FPG, 7,328 OGTT and 55,279 HbHbA1c. 1119 subjects were simultaneously submitted for the three laboratory tests (HbA1c, FPG and OGTT), six individuals were excluded as they were <18 years (S1 Supporting information). The mean age was 53.8 ± 15.2 years (range 18–97 years) and 67.1% were female. The mean HbA1c was 5.5 ± 0.58%, mean FPG was 95.0 ± 16.4 mg/dl and mean OGTT was 141.5 ± 76.2 mg/dl. Mean HbA1c, FPG and OGTT showed a linear increase with the age (Wilcoxon rank-sum test, P trend <0.001; Table 1).

**Table 1:** Fasting plasma glucose, or 2-hour post 75-g oral glucose tolerance test, or hemoglobin HbA1c by age group.

	Age						P value
	< 30 yr (n = 84)	30–39 yr (n = 124)	40–49 yr (n = 184)	50–59 yr (n = 285)	60–69 yr (n = 272)	≥70 yr (n = 164)	
**FGP**	83.0±9.4	85.4±11.1	92.4±13.6	96.1±13.1	100.1±21.5	101.1±14.0	<0.001
**OGTT**	95.2±20.6	104.8±36.6	122.5±65.3	140.4±74.1	161.5±83.1	183.1±87.7	<0.001
**HbA1c**	5.0±0.3	5.2±0.3	5.4±0.6	5.6±0.5	5.7±0.6	5.9±0.7	<0.001

Wilcoxon rank-sum trend test, p<0.05

**Fig 1:**
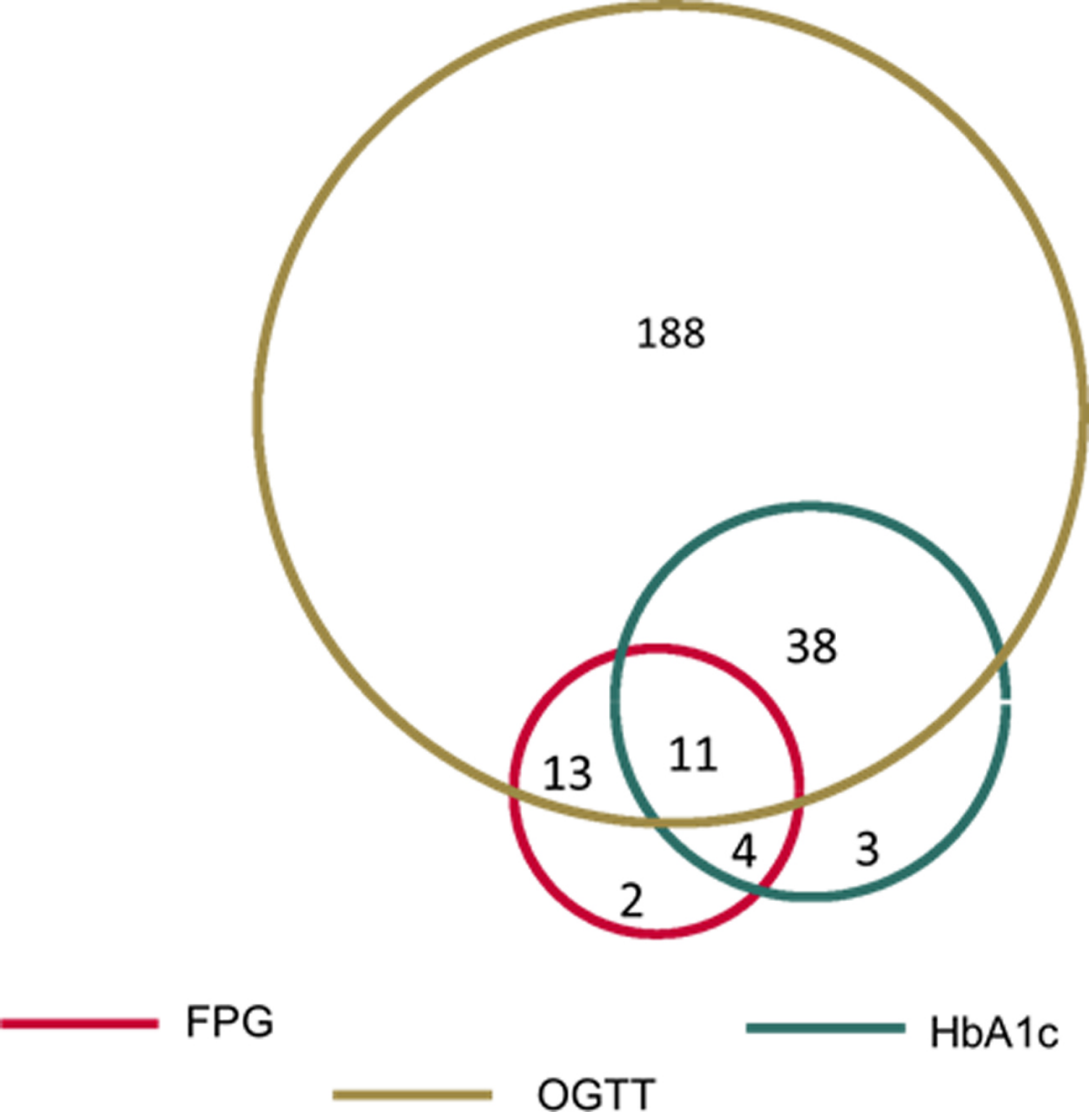
Diagnosis of DM2 (N = 259) by fasting plasma glucose (FPG), glycosylated hemoglobin (HbA1c), oral glucose tolerance test (OGTT), and by combinations of these tests.

259 subjects [(23.3%; 95% CI: 20.7–25.8%] were diagnosed as diabetic according to the ADA guidelines of a positive value (above cut-point) in any single test. 30 subjects (2.7%) were identified as diabetic by FPG, 56 subjects (5.0%) by HbA1c, and 250 subjects (22.5%) by OGTT (Fig 1). Of the 259 subjects diagnosed as DM2, 30 (11.6%) individuals were identified by FPG (≥ 126 mg/dl), 56 individuals (21.6%) by HbA1c (≥ 6.5%) and 250 individuals (96.5%) by OGTT (≥200 mg/dl). Table 2 compares number/%, classification of normal, elevated risk for (prediabetes) [10–12], and DM2 according to each test type, taking OGTT as a reference on the basis that it identified more patients with DM2. 20% of individuals that were identified as DM2 by OGTT, were normal according to cut-off points for FPG and 12% by HbA1c cut-off points. The weighted Cohen's kappa showed a low agreement of FPG and HbA1c with OGTT.

**Table 2:** Number/% classification of normal, risk for (prediabetes), and DM2 according to each test type, but OGTT as a reference.

	OGTT		
	Normal <140 mg/dl	Risk for (prediabetes) 140–199 mg/dl	Diabetes ≥ 200 mg/dl
**FGP**			
**Normal < 100 mg/dl**	633 (84.6%)	34 (4.6%)	81 (10.8%)
**Prediabetes 100–125 mg dl**	167 (49.8%)	23 (6.9%)	145 (43.3%)
**Diabetes ≥ 126 mg/dl**	3 (10.0%)	3 (10%)	24 (80.0%)
**HbA1c**			
**Normal < 5.7%**	615 (86.9%)	37 (3.8%)	66 (9.3%)
**Prediabetes 5.7–6.4%**	185 (53.0%)	29 (8.3%)	135 (38.7%)
**Diabetes ≥ 6.5%**	3 (5.4%)	4 (7.1%)	49 (87.5%)

Weighted Cohen's Kappa (OGTT vs FPG): 0.299 ± 0.021

Weighted Cohen's Kappa (OGTT vsHbA1c): 0.365 ± 0.022

## Discussion

We evaluated data collected in 1113 adults who were referred for DM2 screening and undertook FPG, OGTT and HbA1c tests simultaneously for the diagnosis of DM2 [5,6]. Diagnosis of DM2 within the present population increased with age independent of the test used, aligning with previous evidence that age is an important risk factor for the disease [7]. The OGTT identified the largest number of individuals as diabetic (22.5% of the sample) according to the ADA guidelines. In contrast, only 2.7% of the sample were diagnosed as diabetic by FPG, and 5% by HbA1c and detection was only increased by 0,8% when all three tests implemented compared to OGTT alone. Moreover, of the subjects diagnosed with DM2 by OGTT, less than 10% also had FPG or HbA1c values that classified them as DM2. Our findings suggest that routine screening using all three tests with the ADA cut points does not appear to be appropriate in the Colombian adult population given the very marginal increase in detection capacity achieved with this approach. Instead, using a combination of FPG and OGTT (cost $ 3.58 USD) in place of the 3 tests (cost $ 10.55 USD) for screening would have saved $7811 USD and yet have only missed 3 (of 259) patients who were identified only by HbA1c (cost $ 6.98 USD) and not by OGTT or FPG. Subsequently assessing HbA1c in patients identified as DM2 as a baseline for evaluating the impact of treatment, would add a cost of $ 1787, but there would still be a saving of $6042 relative to using the three tests for screening.

Indeed, until 2010, FPG and OGTT were the tests routinely used to diagnose DM2, at which point, based on the recommendation of several organizations an HbA1c of ≥ 6.5% was introduced as an additional diagnostic criterion [13–16]. While several prospective cohort studies suggested that this test was a convenient and effective method for screening DM2 [17–20], several other studies in diverse populations have disputed the utility of HbA1c based on its similar sensitivity and specificity as FPG in screening for new cases of DM2 and its inferiority to the OGTT as a confirmatory test [21–25]. In addition, significant differences in HbA1c cut-off points for DM2 according to ethnicity, age, sex, and population prevalence of diabetes were noted [7,8]. In US adults Karnchanasorn et al [9] reported also found that HbA1c ≥ 6.5% had a low diagnostic capacity relative to both FPG and OGTT, suggesting that a substantial number of cases of DM2 would be missed using this criterion. This data, from over 5000 participants of the NHANES 2005–2010 showed that of 392 subjects who met ADA criterion for DM2 according to OGTT only 28.1% were identified by HbA1c, while of 245 subjects who met FPG cut points only 43.3% were also diagnosed by HbA1c. The authors concluded that “the HbA1c cut-off value should be revised for better sensitivity to better identify individuals in an early diabetic state”. The present analysis broadly aligns with these findings in confirming the poor diagnostic capacity of HbA1c in Colombian adults, although we noted larger discrepancies between the sensitivity of OGTT versus the two other tests than they did. This may be partly related to the older age of our population (mean age 54) compared to the HNANES study (mean age 46), and age being associated with a progressively larger increment in OGTT than FPG or HbA1c values.

HbA1c does however have the practical advantage over the OGTT and FPG in that it can be performed on a non-fasting blood sample, reducing scheduling pressure on laboratories in the early morning, and potentially making it more convenient for patients. Moreover, patients who did not follow the fasting instructions for OGTT and FPG, would need to return another day. These factors combined with the recent Colombian diabetes guidelines [5] recommending that one or more of the three criteria could be used to diagnose DM2, may have led to physicians routinely requesting all three tests when there is clinical suspicion of DM2. Our findings demonstrate that this approach does not improve the detection, while it does increase the cost to the patient or the health system.

Our study, based on the cross-sectional analysis of a database of a large local laboratory has several limitations, including lack of anthropometric data and risk factors, clinical reasoning behind the decision to send the patient for screening with the three tests. We have previously validated a screening tool—the FINDRISC—in Colombian adults [26,27], which could provide a standardized and low cost means to identify elevated risk individuals for whom laboratory screening tests are warranted. Another study limitation is the lack of follow-up information on the clinical progress of the subjects and potential vascular complications. Furthermore, there is no external gold standard measures by which to evaluate the sensitivity and specificity of the each of the three tests as the ADA criteria for DM2 is meeting the cut-point in any one of the three tests. We therefore only reported on the detection of each test relative to OGTT which was the most sensitive based on current cut-points.

However, the present study is the first conducted in a low-middle income country that has evaluated the diagnostic efficacy of concurrent implementation of FPG, OGTT and HbA1c. There is currently no universal agreement on which is the “best” single test for detecting DM2, however the present study demonstrates that at least in the Colombian population, the OGTT appears to be the most sensitive test for diagnosing DM2 as it detects significantly more cases than FPG or HbA1c. We suggest that there may be a need to reconsider the cut-points for HbA1c in our population and that while HbA1c’s value is recognized for monitoring those with known DM2, the cost of routinely screening with this test is not matched by its efficacy and at least in the present population a significant saving would be made by routine instead using FPG and OGTT, and reserving HbA1c for those who have been identified with DM2.

## Supporting information

S1 Supporting Information Database of the study population including 1119 subjects and 6 variables. (XLSX)
